# Relationship between leg lean mass Z-score and cardiac output at exercise as measured by exercise cardiac magnetic resonance imaging

**DOI:** 10.1186/1532-429X-17-S1-P208

**Published:** 2015-02-03

**Authors:** Kevin K Whitehead, Catherine M Avitabile, David J Goldberg, Mary B Leonard, Zhenglun (Alan) Wei, Elaine Tang, Stephen M Paridon, Ajit P Yoganathan, Mark A Fogel

**Affiliations:** Children’s Hospital of Philadelphia, Sewell, PA USA; Engineering, Georgia Institute of Technology, Atlanta, GA USA

## Background

We previously showed that leg lean mass Z-score (LLMZ) correlates with metabolic exercise performance in Fontan patients. However, the mechanism by which leg lean mass influences exercise is not clear since LLMZ does not correlate with ventricular function or cardiac output at rest. We hypothesized that LLMZ would correlate with cardiac output at exercise and the change in cardiac output from rest to exercise.

## Methods

Thirteen patients had leg lean mass measured by dual energy x-ray absorptiometry within mean of 40 (range 0-258) days of completing an exercise cardiac magnetic resonance (CMR) protocol. LLMZs were generated from healthy reference data. Ventricular volumes and phase contrast flow measurements (all indexed to body surface area) of the ascending (Ao) and descending (DAo) aorta, and superior vena cava (SVC) were obtained by CMR at rest and just after supine ergometer exercise to a heart rate associated with anaerobic threshold (determined by previous metabolic exercise test). Change in systemic flow (Qs = SVC + DAo) and indexed ventricular output (CI) during exercise, as well as baseline and peak exercise measures of Qs and CI were compared to LLMZ by linear regression.

## Results

There was no correlation between LLMZ and resting flows. However, there was a strong linear relationship between LLMZ and change in both CI and Qs from rest to exercise (see figure). There was also a significant correlation between LLMZ and Qs at exercise (r=0.70, p=0.008). The correlation between LLMZ and CI at exercise did not reach significance (r=0.53, p=0.07).

**Figure 1:**
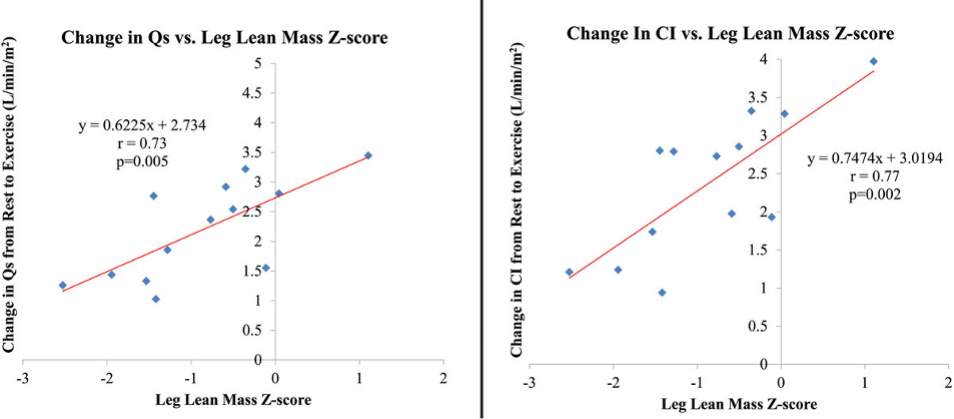
Figure 1

## Conclusions

In our cohort, there was a strong correlation between LLMZ and change in both CI and Qs from rest to exercise. This suggests Fontan patients with higher LLMZ are better able to augment CI during exercise, improving performance. While it is tempting to attribute these differences to the effects of a peripheral muscle pump, cardiac output was not measured during active exercise but immediately after exercise. A prospective study will be needed to determine the effect of lower extremity strength training on cardiac output at exercise and exercise performance.

## Funding

This research was supported by NIH National Heart, Lung, and Blood Institute grants K23 HL089647 (KKW) and R01 HL67622.

